# EBV LMP1-C terminal binding affibody molecule downregulates MEK/ERK/p90RSK pathway and inhibits the proliferation of nasopharyngeal carcinoma cells in mouse tumor xenograft models

**DOI:** 10.3389/fcimb.2022.1078504

**Published:** 2023-01-04

**Authors:** Yanru Guo, Saidu Kamara, Jing Zhang, He Wen, Maolin Zheng, Ying Liu, Luqi Zhou, Jun Chen, Shanli Zhu, Lifang Zhang

**Affiliations:** Institute of Molecular Virology and Immunology, Department of Microbiology and Immunology, School of Basic Medical Sciences, Wenzhou Medical University, Wenzhou, Zhejiang, China

**Keywords:** EBV, LMP1, affibody molecules, nasopharyngeal carcinoma, targeted therapy

## Abstract

Nasopharyngeal carcinoma (NPC), is an Epstein-Barr virus (EBV) associated malignancy most common in Southern China and Southeast Asia. In southern China, it is one of the major causes of cancer-related death. Despite improvement in radiotherapy and chemotherapy techniques, locoregional recurrence and distant metastasis remains the major causes for failure of treatment in NPC patients. Therefore, finding new specific drug targets for treatment interventions are urgently needed. Here, we report three potential Z_LMP1-C_ affibody molecules (Z_LMP1-C_15, Z_LMP1-C_114 and Z_LMP1-C_277) that showed specific binding interactions for recombinant and native EBV LMP1 as determined by epitope mapping, co-localization and co-immunoprecipitation assays. The Z_LMP1-C_ affibody molecules exhibited high antitumor effects on EBV-positive NPC cell lines and displayed minimal cytotoxicity towards EBV-negative NPC cell line. Moreover, Z_LMP1-C_277 showed higher antitumor efficacy than Z_LMP1-C_15 and Z_LMP1-C_114 affibody molecules. The ability of Z_LMP1-C_277 decrease the phosphorylation levels of up-stream activator phospho-Raf-1^(Ser338)^, phospho-MEK1/2^(Ser217/Ser221)^, phospho-ERK1/2^(Thr202/Thr204)^, thereby leading to downstream suppression of phospho-p90RSK^(Ser380)^ and transcription factor c-Fos. Importantly, tumor growth was reduced in tumor-bearing mice treated with Z_LMP1-C_277 and caused no apparent toxicity. Taken together, our findings provide evidence that Z_LMP1-C_277 as a promising therapeutic agent in EBV-associated NPC.

## Introduction

Epstein- Barr virus (EBV), a human herpesvirus, is a widely prevalent pathogen in human and is carried by most individual as a latent and asymptomatic infection 90-95% of the world’s adult population (Katano, 2010). EBV has been linked to the development of numerous human malignancies, including nasopharyngeal carcinoma (NPC), endemic Burkitt’s lymphoma, Hodgkin’s lymphoma and gastric cancer (Young et al., 2016). More recently, there has been growing interest to investigate the relationship between EBV infection and NPC initiation. Many molecular and serological data has demonstrated the crucial role of EBV in NPC pathogenesis. Serum antibodies to EBV can be detected in nearly all cases of undifferentiated or poorly differentiated NPC patients, as well as high expression levels of viral miRNAs in NPC biopsy samples (Wolf and Becker, 1973; de Schryver et al., 1969; Pathmanathan et al., 1995). EBV-associated NPC is a head and neck neoplasm common in Southern China and Southeast Asia (Torre et al., 2015). Due to development in radiotherapy techniques, advances in surgery and the wide application of chemotherapy, greatly improved the quality of life and survival outcomes for patients with NPC (Zhou et al., 2017). However, distance metastasis, disease recurrence and advanced local tumors remain poor prognostic factor (Zhou et al., 2017). At initial diagnosis, more than 80% of NPC patients are diagnosed in advanced stages or metastatic disease (III and IV), which linked to reduce survival rate (Liu et al., 2017). Therefore, there is an urgent need to identify new targets and develop effective therapies to treat EBV-associated NPC.

In NPC, latent EBV genome express five-encoded nuclear antigens (EBNA) and two latent membrane proteins (LMP1 and LMP2) (Dawson et al., 2012; Young and Dawson, 2014). The EBV-encoded latent membrane proteins play an important role in NPC etiology by engaging of multiple signaling pathways which collectively promotes cell proliferation, apoptosis, migration and invasion (Scholle et al., 2000; Dawson et al., 2012; Young and Dawson, 2014). LMP2 could contribute a role in maintaining viral latency in EBV infected B cells (Dawson et al., 2012). Unlike LMP2, LMP1 is critical for EBV-induced transformation in human B cells (Luftig et al., 2004). LMP1 contains three domains, a short N cytoplasmic tail of 23 amino acids, transmembrane domain of 164 amino acids and a long carboxy-terminal tail of 200 amino acids (Tsao et al., 2002). The long carboxy-terminal region constitutes most of the cell signaling proteins, which contains three distinct functional domains referred to as C-terminal activating regions 1, 2 and 3 (CTAR1, CTAR2 and CTAR3). All of these domains are essential for protein-protein interaction that regulate a variety of signal transduction pathways, which include ERK-MAPK, JAK/STAT, PI3-K/Akt and NF-κB JNK/p38-SAPK, to promote NPC tumor growth and invasion (Tsao et al., 2002; Li and Chang, 2003; Morris et al., 2009). CTAR-1 contains a PxQxT motif that binds to TNFR-associated factor (TRAFs), whereas CTAR-2 contains a YYD motif that binds to TNFR-associated death domains (TRADD) (Kim et al., 2000; Mainou et al., 2005; Morris et al., 2009). These two activation regions are often responsible for translating signals, leading to differential gene expression associated with cell cycle progression and matrix metalloproteinase 7 (MMP7) (Dawson et al., 2012; Young et al., 2016). In addition, LMP1 is detected in approximately 60% of NPC patients, while mRNA expression of LMP1 is detected in 91% of NPC tissue samples using RT-PCR (Fåhraeus et al., 1988). Therefore, LMP1 represents a good therapeutic target in the treatment of EBV-associated NPC.

Affibody molecules are a class of small (6.5 kDa) non-immunoglobulin affinity proteins generated by combinatorial library of the three-helix scaffold of the Z domain derived from staphylococcal protein A (Löfblom et al., 2010). The three-helix bundle structure has been prepared as scaffold for construction of combinational libraries, by the concerted randomization of amino acid sequence, from which desired target proteins can be selected using phage display method (Nord et al., 1995). Their small size exerts deep tumor penetration as well as ideal pharmacokinetics (i.e., fast blood clearance) for use as diagnostic and treatment agents, as compared to antibodies (Löfblom et al., 2010; Ståhl et al., 2017). Recently, over 400 studies have been reported in which affibody molecules have been generated and used in a variety of applications (Ståhl et al., 2017). The affibody molecules targeted proteins include epidermal growth factor (EGFR) (Andersson et al., 2016; Oroujeni et al., 2018), human epidermal growth factor 2 (HER2) (Orlova et al., 2006; Sörensen et al., 2016), human epidermal growth factor 3 (HER3) (Malm et al., 2013; Schardt et al., 2017), vascular endothelial growth factor (VEGF) (Fedorova et al., 2011), human papilloma virus type 16 E7 (HPV16 E7) (Xue et al., 2016; Zhu et al., 2018), LMP2A-N (Zhu et al., 2020), and LMP1-C (Kamara et al., 2021), offer a great potential in diagnosis and therapy.

In our previous study (Kamara et al., 2021), we generated three novel LMP1-C terminal domain binding affibody molecules (Z_LMP1-C_15, Z_LMP1-C_114 and Z_LMP1-C_277) and their ability to bind to recombinant and native LMP1-C terminal protein were investigated both *in vitro* and *in vivo*. In this study, we explored the antitumor effects of Z_LMP1-C_ affibody molecules on EBV-positive NPC cell lines, and further elucidate the inhibitory effects of Z_LMP1-C_277 on MEK/ERK/p90RSK signaling pathway in NPC cells. Moreover, Z_LMP1-C_277 for targeted therapy of NPC in tumor-bearing nude mice.

## Materials and methods

### Animals, cells and vectors

Female BALB/c nude mice, 6-8 weeks old, weighing 18 g, purchased from Shanghai SLAC Laboratory Animal Co. Ltd. All the animals were house under controlled laboratory conditions at the animal facility of Wenzhou Medical University. The animal experiments were performed in accordance with the rules of animal Ethical Committee of Wenzhou Medical University. C666-1 and CNE-2Z (NPC positive) cells were purchased from Taisheng Bio-Tech Guangzhou, China Co., Ltd and HNE-2 (NPC negative) from Shanghai Fu life Industry Co., Ltd. The cells were cultured as previously described (Xue et al., 2016). Plasmid *p*ET21a (+) as protein expression vector and bacterial host strain *Escherichia coli* BL21 (DE3) were purchased from Novagen and ATCC, respectively.

### Protein expression and purification

A phage display of combinatorial library was constructed in our laboratory, from which three affibody molecules that bind with high affinity and specificity to EBV LMP1-C were selected (Kamara et al., 2020). The Z_LMP1-C_ affibody molecules were selected for further study. The expression of Z_LMP1-C_ and Z_WT_ affibody molecules (unselected original affibody scaffold molecule with no binding affinity to LMP-1 used as negative control) were induced by 1 mM IPTG (Sigma Aldrich, Saint Louis, USA) and purified with Ni-NTA Agarose (Qiagen, Valencia, CA) according to the manufacturer’s instructions. Proteins were analyzed by sodium dodecyl sulfate-polyacrylamide gel electrophoresis (SDS-PAGE) and detected by Western blotting using anti-His antibody (MultiSciences Biotech Hangzhou, China Co., Ltd). Then, measurement of protein concentration using Bicinchoninic Acid (BCA) protein assay kit and stored at -80°C for future use.

### Epitope mapping by ELISA

The peptides (Shanghai Bootech Bioscience &Technology Co., Ltd) were dissolved in coating buffer (10μg/ml) and immobilized on 96-well microtiter plates at 4°C overnight. After plates incubated overnight, they were blocked with 5% skim milk powder (0.1% Tween 20 in phosphate-buffered saline) and were then treated with Z_LMP1-C_ affibody molecules (100μg/ml). The plates were washed and probed with anti-His-tag-mouse IgG and then incubated with HRP conjugated anti-mouse IgG. Next, add 100µl of TMB substrate first, and 50 µl stop solution to each well. Measure the absorbance at 450 nm with a microplate reader (BioTek, Winooski, VT, USA). All tests were run in triplicate, and EBV LMP1-C polyclonal antibody was used as positive control.

### Cell culture

EBV-positive NPC cell lines (C666-1 and CNE-2Z) were obtained from (Taisheng Bio-Tech Guangzhou, China Co., Ltd) and EBV-negative NPC (HNE-2) (Shanghai Fu life Industry Co., Ltd). C666-1 and CNE-2Z cells harbor EBV DNA and express LMP1 protein and HNE-2 EBV-DNA negative were used to evaluate Z_LMP1-C_ affibody molecules binding affinity and antitumor effects. Cell lines were cultured in RPMI-1640 and DMEM media (Gibco-BRL, Gaithersburg, MD, USA) supplemented with 10% fetal bovine (FBS) and penicillin/streptomycin 100 units/mL and 0.1 mg/mL (Gibco-BRL, Gaithersburg, MD, USA).

### LMP1 expression level in NPC cells

Briefly, C666-1, CNE-2Z and HNE-2 were cultured and total RNA was extracted using TRIzol reagent Takara Biomedical Technology (Beijing) Co., Ltd. Then, the RNA was reverse transcribed into cDNA. Samples were mixed with primers (1. LMP1:5’-TCATCGCTCTCTGGAATTTG 3’TAAGCAGCCAAAGATGAACA;2. GAPDH: 5’-TGAACGGGAAGCTCACTGG, 3’TCCACCACCCTGTTGCTGTA) and using q-PCR master mix for real time PCR (Thermo Fisher Scientific, MA, U-

SA). All data were analyzed using QuantStudio Real-Time PCR software (Life Technologies).

We further examined the expression level in NPC cells and Western blotting was performed. Cells were harvested, rinse in ice-cold PBS and lysed in lysis buffer. For Western blotting, samples were separated by 15% SDS-PAGE, and transblotted to polyvinylidene difluoride (PVDF) membranes. After, membrane was blocked with 5% skim milk in PBS containing 0.1% Tween 20 (PBST) for 2h at 37°C. Add primary antibody anti-LMP1 (Abcam 136633) store at 4°C overnight, and then incubate with secondary antibody. Bands were visualized by Western blotting Imaging System (Clinx, Shanghai, China). Glyceraldehyde 3-phosphate dehydrogenase (GAPDH) serves as internal control.

### Confocal microscopy

C666-1 cells were seeded on a 6-well plate, culture in 5% CO2 incubator at 37°C. After 24 h, the medium was replaced with a fresh media containing 100μg/mL of Z_LMP1-C_ or Z_WT_ affibody molecule and store for 3 h at 37°C. Treated cells were fixed with 4% paraformaldehyde and permeabilized with 0.3% Triton X-100 for 10 min at 37°C. Cells were blocked with blocking buffer (1% BSA) at 37°C for 1h and then incubated with primary antibody (mouse anti-His-tag mAb), and LMP1 rabbit mAb followed by secondary antibodies FITC-conjugated goat anti-mouse IgG (H+L) and CY3 Ab rabbit (diluted in blocking buffer) for 1 h at 37°C. Then, cells were counterstained with Hoechst 3342 and imaged using confocal microscopy (Nikon C1-i, Japan).

### Immunoprecipitation

C666-1 cells were cultured for 24 h, after which Z_LMP1-C_ or Z_WT_ (control) affibody molecule were added to a final concentration of 100 μg/mL for 3 h at 37°C. After three washings with PBS, cells were lysed with RIPA lysis buffer supplemented with protease inhibitors. The rabbit antibody specific for LMP1 (Abcam) combined with disuccinimidyl suberate bound to protein A/G plus agarose (Beyotime, Beijing, China) were used. Next, the protein complexes were resuspended in SDS sample buffer and subjected to Western blotting.

### *In vitro* efficacy of Z_LMP1-C_ affibody molecules

Cell Counting Kit-8 (CCK-8) assays were employed to study the efficacy of Z_LMP1-C_ affibody molecules in EBV-positive NPC cell lines. C666-1, CNE-2Z and HNE-2 cells were seeded onto a 96-well microtiter plates at a density of 5x10^3^ cells per well and then treated with Z_LMP1-C_ affibody molecules at different concentrations from 0.6, 1.2, 2.5, 5.0, 10 and 20 μM. NPC cells treated with Z_WT_ affibody molecule and HNE-2 treated with Z_LMP1-C_ affibody molecules were set as negative control. The cell viability was measured for each concentration and incubation time (12, 24, 36, 48 and 72 h), respectively. Subsequently, 10μL of CCK-8 solution was added per well and incubated for 30min. After adding the kit solution, absorbance at 450nm was measured using microplate reader.

### 5-Ethynyl-2’-deoxyuridine cell proliferation detection

Cell proliferation was detected using EdU assay kit (Beyotime, Beijing, China) according to the manufacturer’s protocol. C666-1, CNE-2Z and HNE-2 were seeded at density of 2.5 x10^4^ cells per well, incubated with100 μg/mL Z_LMP1-C_ or Z_WT_ affibody molecule for 24 h at 37°C. Then, 20μM EdU labelling medium was added to the cell culture for 2 h at 37°C, and the cells were fixed and subsequently stained by Hoechst 33342. For each experiment, three random fields were imaged at 400x magnification and the numbers of EdU-positive cells were counted.

### Clonogenic assay

Cell colony formation assay was employed to evaluate cell proliferation. C666-1, CNE-2Z and HNE-2Z cells were plated into 6-well culture plates at a density of 1x10^3^ cells per well and then treated with Z_LMP1-C_ or Z_WT_ affibody molecule for 14 days. Next, cells were washed with pre-cooled PBS, fixed with 4% paraformaldehyde and then stained with 0.1% Crystal Violet Staining Solution (Amresco, Solon, OH, United States). The number of colonies greater than 50 were counted under a microscope.

### Flow cytometry analysis

C666-1, CNE-2Z and HNE-2 cells were cultured and harvested after incubation with Z_LMP1-C_ or Z_WT_ affibody molecule for 24 h at 37°C. After treatment, the cells were washed in PBS and fixed with 70% ethanol overnight at 4°C. Then, stained with propidium iodide (50μg/mL) and treated with RNase A (50 U/mL) for 30 min at 37°C in darkness. Cell cycle distributions were determined by a FACS Calibur flow cytometer (BD, Biosciences, San Jose, CA, USA). Data was analyzed using ModFit LT, Version 3.0 software.

### Signaling proteins analysis by Western blotting

To evaluate the role of MEK/ERK/p90RSK pathway in NPC cell proliferation, we then examined the effects of Z_LMP1-C_277 on NPC cells growth. EBV-positive NPC cells (C666-1 and CNE-2Z) were cultured at 6-well plate at a cell density of (1 × 10^5^) and treated with fresh medium containing either Z_LMP1-C_277 or Z_WT_ affibody for 36 h at 37°C. After 36 h treatment, the cells were lysed for 15 min on ice in lysis buffer supplemented with protease and phosphatase inhibitors and the total protein was quantified using a BCA protein assay kit. Proteins were subjected to 15% SDS-PAGE and transferred onto a PVDF membrane. The membrane was blocked with 5% skim milk and incubated with primary antibodies (**Supplementary Table S1**) at 4°C overnight. Afterwards, the membranes were washed and incubated with secondary antibodies for 2 h at 37°C. Membranes were visualized by Western blotting Imaging System. GAPDH served as an internal reference.

### *In vivo* antitumor efficacy of Z_LMP1-C_277 affibody molecule

In order to investigate the therapeutic performance of Z_LMP1-C_ terminal domain binding affibody towards EBV-positive NPC cell lines, we selected Z_LMP1-C_277 affibody molecule for further study. The nude mice were randomly divided into 5 groups (n= 5 per group). After, nude mice were inoculated with 1×10^7^ EBV-positive and EBV-negative NPC cells suspended in 0.1 mL PBS into the right forelimb by subcutaneous injection. The tumor sizes and body weight of nude mice were recorded every 3 days for 30 days. Once tumors become palpable, tumor growths were serially measured with calipers and tumor volumes calculated using the following formula: volume = length × width^2^ × 0.52. When the tumor reached an average size (50-100mm^3^), mice were treated *via* tail vein at 0.1 mL of Z_LMP1-C_277 (100 nmol/kg), Z_WT_ (100 nmol/kg) cisplatin (5mg/kg) or PBS, respectively. Mice were treated every three days for a total of six doses. The therapeutic efficacy and toxicity of Z_LMP1-C_277 was monitored based on daily measurements of tumor volume and body weight. On day 30, all mice were sacrificed, and the tumors were carefully removed, weighed and stored in -80°C.

### Statistical analysis

The data are presented as the mean ± standard deviation (SD). Statistical analysis of the results was performed using Student’s t-test, and a probability (p) value < 0.05 was regarded as statistically significant. All analysis were performed using GraphPad Prism software.

## Results

### Expression and identification of the binding sites of Z_LMP1-C_ affibody molecules

**Figure 1:**
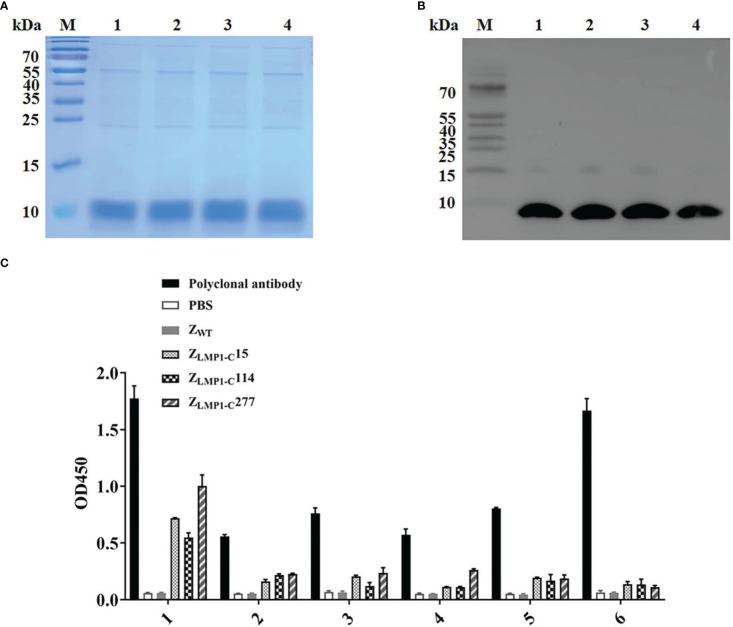
Analysis of binding of Z_LMP1-C_ to recombinant LMP1-C. The purified Z_LMP1-_C affibody molecules were used for SDS-PAGE analysis **(A)** and confirmed by Western blotting **(B)** M, protein marker; 1, Z_LMP1-C_15; 2, Z_LMP1-C_114; 3, Z_LMP1-C_277; 4, Z_WT_. **(C)** Epitope mapping of the Z_LMP1-C_ affibody molecules by ELISA. The truncated peptides (1-6) derived from LMP1-C were designed and synthesized. Both Z_LMP1-C_ and polyclonal antibody reacted with the LMP1-C peptides. In sequence, red font indicates the binding region recognized by Z_LMP1-C_ affibody molecules (**Table 1**). The error bars represent the standard deviation.

As described previously (Kamara et al., 2020), three potential Z_LMP1-C_(Z_LMP1-C_15, Z_LMP1-C_114, and Z_LMP1-C_277) were selected based on ELISA assay, as well as subsequent targeted binding studies using surface plasmon resonance (SPR), indirect immunofluorescence, immunohistochemistry (IHC), and *in vivo* tumor imaging. The recombinant Z_LMP1-C_affibody molecules were expressed in *E.coli* as a His6-tagged fusion proteins, purified by Ni-NTA affinity chromatography, and the purity of the separated proteins were 95% (**Figure 1A**). Moreover, Western blotting result showed that the fusion proteins were detected using the His6-tagged monoclonal antibody (**Figure 1B**).

**Table 1:** Overlapping peptides used for mapping the LMP1-C region.

Number	Peptide Sequence	Domain
1	HDDSLPHPQQATDD	CTAR1
2	GPHDPLPHSPSDSGNDG	CTAR3
3	SDSAGNDGGPPQLTEEVENK	CTAR3
4	GHGGGDPHLPTLLLGSSG	CTAR2
5	LPTLLLGSSGGSGGDDDDH	CTAR2
6	GGDDDDPHGPVQLSYYD	CTAR2

Epitope mapping by ELISA was used to detect the binding site of Z_LMP1-C_ affibody molecules on LMP1-C. The epitope mapping of the Z_LMP1-C_ was performed using 10-mer overlapping peptides derived from LMP1-C translated nucleotide sequence (amino acid, 186-387). Our results indicate that Z_LMP1-C_ affibody molecules could bind to peptide (1). In addition, the Z_LMP1-C_ affibody molecules did not react with other peptides and positive control (anti-LMP1-C polyclonal rabbit antibody prepared in our lab) was similar. Most peptides did not bind to Z_WT_ (affibody negative control). Sequence alignment revealed that the reactive peptide (1) is essential for protein-protein interaction that regulate a variety of signal transduction pathways, which suggested that the Z_LMP1-C_ affibody molecules may bind to the functional region in LMP1-C (**Figure 1C**).

### Intracellular interaction of Z_LMP1-C_ affibody molecules and LMP1

**Figure 2:**
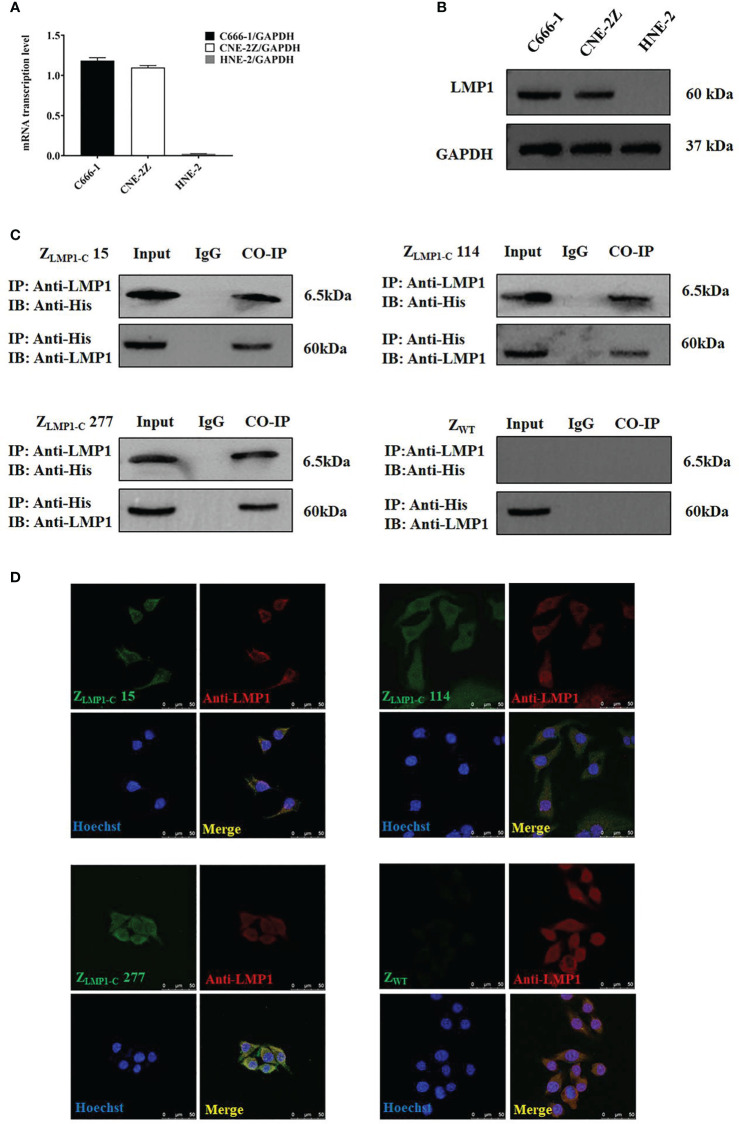
Analysis of intracellular protein. **(A)** RT-PCR analysis of LMP1/GADPH in C666-1, CNE-2Z and HNE-2. **(B)** LMP1expression level in C666-1, CNE-2Z and HNE-2 cells by Western blotting assay. Data are given as mean ± SD (n=3). **(C)** Z_LMP1-C_ affibody molecules complexed with LMP1, then an IP was performed with an anti-LMP1 antibody. Western blotting was performed and incubated with rabbit anti-LMP1 mAb or mouse anti-His-tag mAb. IgG served as a negative control. **(D)**. EBV-positive NPC cell lines (C666-1) were treated with Z_LMP1-C_ or Z_WT_ affibody molecule for 3 h and images were collected by confocal microscopy. Rabbit anti-LMP1 mAb recognized LMP1 and anti-His-tag-mouse mAb are the primary antibodies that were used. Goat anti-rabbit IgG antibody, Cy3 (red) conjugate and goat anti-mouse IgG antibody, FITC Conjugated (green) were used as secondary antibodies. Blue are cell nuclei stained with Hoechst 3342 and yellow are the merge images that showed the co-localization between Z_LMP1-C_ affibody molecules and anti-LMP1. Scale bar = 50 μM.

To further study the binding of Z_LMP1-C_ affibody molecules to the intracellular LMP1, co-localization and co-immunoprecipitation assays were used. Firstly, we measured the expression level of LMP1 in EBV-positive NPC cell lines (C666-1and CNE-2Z) and EBV-negative NPC cell line (HNE-2). The protein expression level of LMP1 was higher in EBV-positive NPC cell lines compared with EBV-negative NPC cell line (**Figure 2A**). The RT-PCR results were confirmed by Western blotting analysis (**Figure 2B**).

As shown in (**Figure 2C**), Z_LMP1-C_ affibody molecules complexed with endogenously expressed LMP1 protein, then an IP was performed with an anti-LMP1 antibody. Z_WT_ was set as affibody negative control. Furthermore, confocal microscopy revealed the co-localization between Z_LMP1-C_ affibody molecules and anti-LMP1. As expected, Z_WT_ did not showed any fluorescence signal (**Figure 2D**). Collectively, co-localization and co-immunoprecipitation results suggest that Z_LMP1-C_ affibody molecules could interact with intracellularly-expressed LMP1 protein.

### *In vitro* efficacy of Z_LMP1-C_ affibody molecules

**Figure 3:**
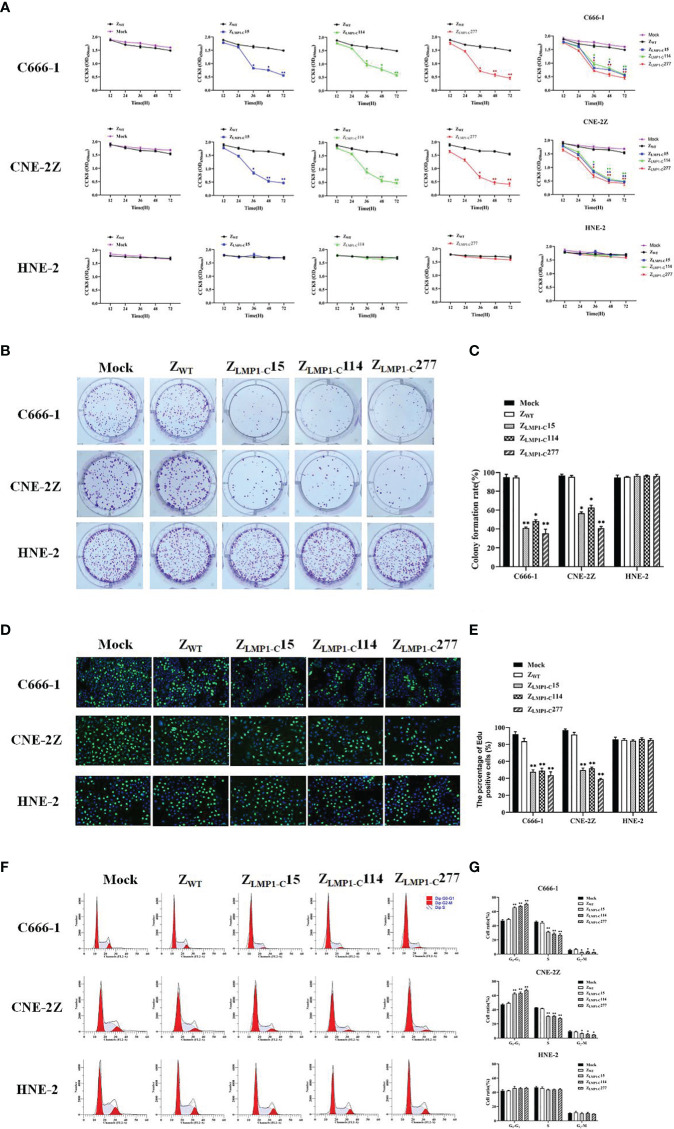
Z_LMP1-C_ affibody molecules significantly inhibited NPC cells proliferation. CCK-8 assays were performed to determine the effects of Z_LMP1-C_ affibody molecules on C666-1, CNE-2Z and HNE-2. **(A)** EBV-positive NPC cell lines incubated with Z_LMP1-C_ affibody molecules reduced the cell viability compared with Z_WT_ control, whereas EBV-negative NPC cell line incubated with Z_LMP1-C_ affibody molecules had no effects on cell viability. **(B)** Colony formation assays were performed for 14 days. The medium and Z_WT_ were set as control groups. **(C)** The graph showed the percentage of colony-forming units after treatment with Z_LMP1-C_ affibody molecules. *P <0.05, **P < 0.01 compared with control. **(D)** C666-1, CNE-2Z and HNE-2 cells were treated with Z_LMP1-C_ affibody molecules for 24 h. Cell proliferation was determined by Edu staining. The images shown. Scale bar = 20 μM. **(E)** Shown are mean values ( ± SD) obtained from three independent repeats. *P <0.05, **P < 0.01. **(F)** Cell cycle analysis with flow cytometry and propidium iodide. **(G)** Cell percentages in G0/G1, S and G2/M phases were calculated from three independents experiments. *P <0.05, **P < 0.01.

In order to prove the efficacy of Z_LMP1-C_ affibody molecules as well as their assessment of cytotoxicity, CCK-8 assays were performed using EBV-positive NPC cell lines (C666-1 and CNE-2Z) and EBV-negative NPC cell line (HNE-2). Cells were incubated for 72 h with increasing concentration of Z_LMP1-C_ affibody molecules. After incubation, viability of EBV-positive NPC cell lines were reduced in a dose-dependent manner (**Supplementary Figure S1**). The half maximal inhibitory concentration (IC50) values of Z_LMP1-C_15, Z_LMP1-C_114 and Z_LMP1-C_277 in C666-1 were 8.588 μM, 8.736 μM, and 3.870 μM, respectively. CNE-2Z IC50 values were 10.914 μM, 6.431 μM, and 9.754 μM, respectively. Then, we selected IC50 value of 10 µM for further studies. We next evaluated the efficacy of Z_LMP1-C_ affibody molecules over the course of 12, 24, 36, 48 and 72 h. As shown in (**Figure 3A**), Z_LMP1-C_15, Z_LMP1-C_114 and Z_LMP1-C_277 affibody molecules effectively reduced the cell viability of EBV-positive NPC cell lines (C666-1 and CNE-2Z) and had no antitumor effects on EBV-negative NPC cell line (HNE-2). Importantly, Z_LMP1-C_277 had better antitumor efficacy than Z_LMP1-C_15, and Z_LMP1-C_114 in both C666-1 and CNE-2Z cells. Cells treated with Z_WT_ affibody remained fully viable. The colony formation assay also proved that long-term treatment with Z_LMP1-C_ affibody molecules caused a decreased of C666-1 and CNE-2Z colonies compared with HNE-2 (**Figure 3B, C**). Moreover, EdU staining were reduced in C666-1 and CNE-2Z cells by treatment with Z_LMP1-C_ affibody molecules, which inhibited DNA replication (**Figure 3D, E**).

To monitor the suppressive effects of Z_LMP1-C_ affibody molecules on cell cycle progression in EBV-positive NPC cell lines, flow cytometry was performed. The representative histograms of the distribution of cell cycle phases in C666-1, CNE-2Z and HNE-2 (**Figure 3F**). Treatment with Z_LMP1-C_ affibody molecules caused an increase accumulation of cells in G0/G1 phase in C666-1 and CNE-2Z, followed by reduction in S and G2/M phases (**Figure 3G**). Overall, these findings support that Z_LMP1-C_ affibody molecules significantly inhibits cell proliferation of EBV-positive NPC cell lines and had no antitumor effects on EBV-negative NPC cell line.

### Z_LMP1-C_277 downregulates MEK/ERK/p90RSK pathway in NPC cells

**Figure 4:**
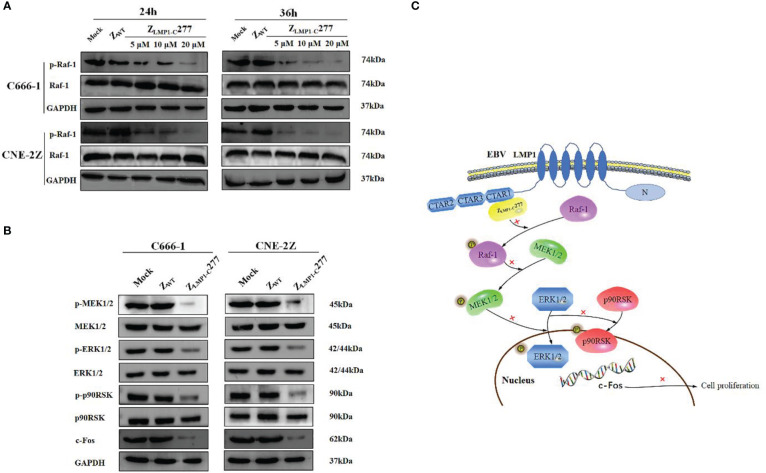
Analysis of MEK/ERK/p90RSK pathway by Western blotting. As shown in **(A)**, Z_LMP1-C_277 treatment induced phospho-Raf-1^(Ser338)^ decrease in a concentration- and time-dependent manner in NPC cells. Western blotting showed that Z_LMP1-C_277 induced a reduction of phospho-MEK1/2 ^(Ser217/Ser221)^, phospho-ERK1/2^(Thr202/Thr204)^, phospho-p90RSK^(Ser380)^ and transcription factor c-Fos levels in NPC cells **(B)**. Schematic diagram of Z_LMP1-C_277 blocking the LMP1-C mediated signaling through MEK/ERK/p90RSK pathway **(C)**.

The conserved essential motif **(**PxQxT) in the CTAR-1 region, is the possible binding site of Z_LMP1-C_ affibody molecules. Then, we focused on MEK/ERK/p90RSK signaling cascade, which is closely linked to CTAR-1 region and growth factor signal transmission, and cell proliferation (Zheng et al., 2007; Burotto et al., 2014; Luo et al., 2021). To investigate whether Z_LMP1-C_277 suppresses NPC cells through MEK/ERK/p90RSK pathway, Western blotting assays was employed. Our results showed that the level of phospho-Raf-1^(Ser338)^ decrease in a concentration- and time-dependent manner in C666-1 and CNE-2Z cells treated with Z_LMP1-C_277 compare to control groups mock and Z_WT_ (**Figure 4A**). According to the above results, we selected 10µM of Z_LMP1-C_277 and treatment time (36 h) to further study the downstream targets of MAP kinase. Western blotting revealed that treatment with 10µM of Z_LMP1-C_277 for 36 h decreased the expression levels of phospho-MEK1/2^(Ser217/Ser221)^, phospho-ERK1/2^(Thr202/Thr204)^, phospho-p90RSK^(Ser380)^ and transcription factor c-Fos in C666-1 and CNE-2Z (**Figure 4B**). Schematic diagram of Z_LMP1-C_277 blocking the LMP1-C mediated signaling through MEK/ERK/p90RSK pathway (**Figure 4C**). However, treatment with Z_LMP1-C_277 did not induce a reduction of phospho-MEK1/2 ^(Ser217/Ser221)^, phospho-ERK1/2^(Thr202/Thr204)^, phospho-p90RSK^(Ser380)^ and transcription factor c-Fos levels in HNE-2 cell line (**Supplementary Figure S2**). Collectively, these findings suggested that Z_LMP1-C_277 inhibited the proliferation of NPC cells by downregulating MEK/ERK/p90RSK signaling pathway.

### Therapeutic efficacy of Z_LMP1-C_277 in NPC-bearing nude mice

**Figure 5:**
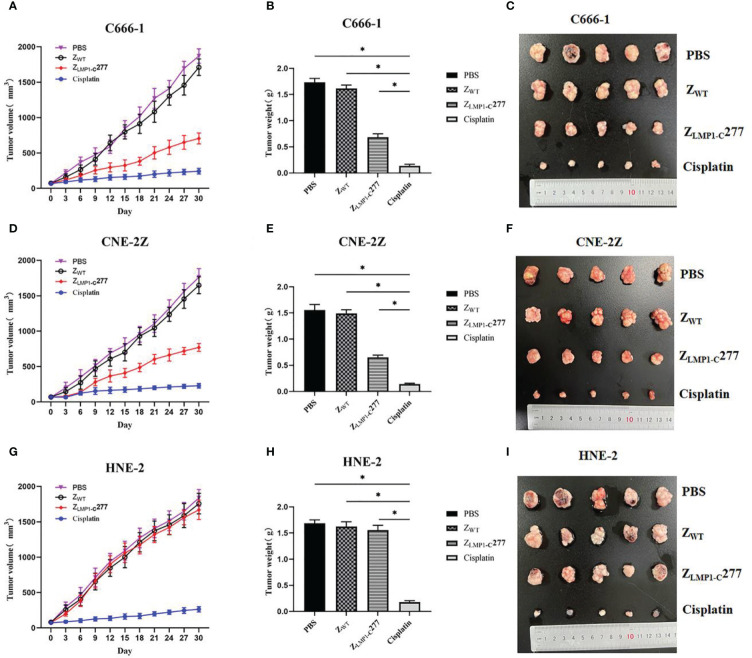
*In vivo* antitumor efficacy of Z_LMP1-C_277. **(A, D)** Tumor volumes of NPC-bearing nude mice after various treatments (PBS, Z_WT_, Z_LMP1-C_277 and cisplatin). **(C, F)** Photographs of the tumors separated from C666-1 and CNE-2Z mice after different treatments. **(G)** Tumor volumes **(I)** tumors separated from mice in HNE-2 group. All tumors from **(B)** C666-1 **(E)** CNE-2Z, and **(H)** HNE-2 were weighed and compared. Data are given as mean ± SD (n=5). *P <0.05 compared to control groups.

*In vivo* antitumor efficacy of Z_LMP1-C_277 was evaluated in C666-1, CNE-2Z and HNE-2 tumor-bearing nude mice by measuring mice weight and calculating tumor volume and tumor growth. As shown in (**Figure 5A–E**), tumors in treated PBS and Z_WT_ (negative controls) mice grew faster than Z_LMP1-C_277 and cisplatin (positive control). Administration of Z_LMP1-C_277 reduced the tumor volume in NPC-bearing nude mice after 15 days of treatment. However, Z_LMP1-C_277 did not inhibit HNE-2 tumor growth, while no apparent sign of toxicity (**Figure 5G–I**). The tumors from the group of Z_LMP1-C_277 showed reduced tumor growth compared to PBS and Z_WT_ groups.

## Discussion

In the treatment of EBV-associated tumors, there are currently few therapies specifically targeted to the oncoproteins within tumors (Kanakry and Ambinder, 2013). Concurrent radiochemotherapy with cisplatin have yielded good cancer control effects in NPC patients (Nie et al., 2019). However, local and regional recurrence remains the predominant cause of treatment failure in NPC patients. Therefore, a better understanding of new EBV targets and pathway is essential for enhancing the radiosensitivity of NPC.

Affibody molecules are engineered affinity proteins, imitating monoclonal antibodies, and are therefore a member of the family of antibody mimetics. Since their first introduction 30 years ago, a series of affibody molecules specific for different targets have been reported. These affinity proteins are highly suited for carrying toxic payloads, blocking protein interactions, and *in vivo* imaging and treatment applications (Nord et al., 1995; Löfblom et al., 2010). According to a recent study, the first candidate of therapeutic affibody (ABY-035) was tested in clinical trials and proven to be safe and well tolerated in healthy volunteers (www.affibody.se). Additionally, their fast folding and simple structure of affibody molecules allow their production by use of conventional peptide synthesis methods, which greatly facilitate production and clinical utilization. In this study, we have expressed and highly purified three potential binding affibody molecules (Z_LMP1-C_15, Z_LMP1-C_114 and Z_LMP1-C_277) in a prokaryotic expression system, and expected molecular weight was further confirmed by using Western blotting. Epitope mapping of Z_LMP1-C_ was performed, which mapped the binding region of these affibody molecules to the EBV LMP1-C terminal domain. Furthermore, the co-localization analysis result implied possibility that there might be interaction between Z_LMP1-C_ affibody molecules and intracellularly-expressed LMP1 protein. More importantly, co-immunoprecipitation confirmed the intracellular protein-protein interactions and has also provided further evidence to support permeation (Moellering et al., 2009). These results showed that Z_LMP1-C_ binding proteins interact specifically with the target protein LMP1.

Monoclonal antibody therapeutics are the most successful and widespread affinity proteins for different life science applications due to their high sensitivity and specificity to any given target (Berger et al., 2002), especially in recent years, anti- programmed cell death protein 1 (PD-1)/programmed death ligand 1 (PD-L1) inhibitors can provide overall survival benefits over conventional therapies for treatment of advanced or metastatic cancers (Sun et al., 2020). Monoclonal antibodies (mAbs) have various mechanisms of action such as target receptor neutralization, signalling disruption, antibody-dependent cell-mediated cytotoxicity (ADCC) and immune checkpoint inhibition (Chames et al., 2009). However, owing to their large macromolecular size (150 kDa), low permeation and susceptibility to degradation limits their use in cancer diagnosis and treatment (Chames et al., 2009). Most recently, affibody molecules are a novel class of small (6.5 kDa) engineered affinity proteins with proven potential in diagnostic and therapeutic applications (Löfblom et al., 2010; Ståhl et al., 2017). Moreover, published study has confirmed that HER2-specific affibody molecules labeled with 111 In have shown to be useful for imaging of HER2 expression in breast cancer and therapeutic applications (Baum et al., 2010). In contrast to antibodies, affibody molecule lack Fc regions; hence, absent of half-life extension and effector function. Therefore, their therapeutic action can be achieved by either blocking ligand-receptor interactions or carrying of toxic payloads (Frejd and Kim, 2017). Considering that, the LMP1-mediated activation of ERK/MAPK, PI3K/AKT, NF-κB, and JAK/STAT signaling pathways, promoting NPC cell proliferation and survival (Tsao et al., 2002; Li and Chang, 2003; Morris et al., 2009), and we then focused on the study of molecular mechanisms of Z_LMP1-C_ treatment that induce an inhibitory effect on NPC cells proliferation. CCK-8, colony formation, and EdU assays demonstrated that Z_LMP1-C_ affibody molecules strongly suppressed NPC cells proliferation. Furthermore, cell cycle analysis showed that treatment of Z_LMP1-C_ affibody molecules increased the cell number G0/G1 phase, and decreased cell number in G_2_/M phase, compared to control groups. Of note, Western blotting showed that Z_LMP1-C_277 affibody molecule induced a reduction in phospho-Raf-1^(Ser338)^, phospho-MEK1/2 ^(Ser217/Ser221)^, phospho-ERK1/2^(Thr202/Thr204)^, phospho-p90RSK^(Ser380)^ and transcription factor c-Fos levels. Our findings confirmed that Z_LMP1-C_277 affibody molecule has the capacity to suppressed NPC cells proliferation, indicating potential therapeutic target for EBV-associated NPC.

*In vivo* antitumor efficacy of Z_LMP1-C_277 affibody molecule was also evaluated. Moreover, the *in vivo* antitumor therapeutic efficacy of Z_LMP1-C_277 showed significant reduction in the tumor growth rate in C666-1 and CNE-2Z cells compared to the control groups (Z_WT_ and PBS), however, was lower than that of cisplatin (positive control). Furthermore, mice did not display any noticeable signs of toxicity after 15 days of treatment with Z_LMP1-C_277 affibody molecule. These data suggest that Z_LMP1-C_277 treatments significantly decreased tumor growth in EBV-positive NPC cell lines, whereas no apparent toxicity. Nevertheless, further studies are needed to evaluate the clinical applicability of Z_LMP1-C_277 affibody molecule. Immunogenicity is an important consideration in the development and approval of biopharmaceuticals. Despite that though, anti-EGFR1 nanobody were shown to have reduce immunogenicity in both preclinical and clinical evaluation (Harmsen and De Haard, 2007). Albumin binding domain (ABD) has been widely used for the modification of therapeutic proteins (Nilsson et al., 1996), and ABD-fused with affibody molecules have demonstrated reduce immunogenicity, which is more important for *in vivo* tumor imaging and treatment.

## Conclusion

In summary, the specific interaction between these three potential Z_LMP1-C_ affibody molecules (Z_LMP1-C_15, Z_LMP1-C_114, and Z_LMP1-C_277) to native LMP1 protein were confirmed by epitope mapping, co-localization and co-immunoprecipitation assays. *In vitro*, the Z_LMP1-C_ binding affibody molecules exhibited significant antitumor efficacy in EBV-positive NPC cell lines, however, Z_LMP1-C_15 and Z_LMP1-C_114 were lower than that of Z_LMP1-C_277. Moreover, we have showed that Z_LMP1-C_277 could inhibits the phosphorylation of Raf protein (up-stream activator of MAP kinase), leading to downstream suppression of 90 kDa ribosomal S6 kinase (p90RSK) and transcription factor c-Fos. Further in vivo study showed that Z_LMP1-C_277 treatments significantly inhibited tumor growth and reduced systemic toxicity. Our results demonstrated that Z_LMP1-C_277 as a promising therapeutic agent for EBV-associated NPC.

## Data availability statement

The original contributions presented in the study are included in the article/**Supplementary Material**. Further inquiries can be directed to the corresponding author.

## Ethics statement

The animal study was reviewed and approved by the ethics committee of Wenzhou Medical University (wydw2021-0352).

## Author contributions

YG and SK conceived and designed experiments, performed experiments, analyzed the data, and drafted the writing of the manuscript. JZ, HW, MZ, YL and LuZ contributed reagents/materials/analysis tools. SZ and JC revised the manuscripts. LiZ conceived and designed experiments, analyzed data, drafted the writing of the manuscript, funding acquisition, and supervised all experiments.
