# Supplementary material for: EBV LMP1-C terminal binding affibody molecule downregulates MEK/ERK/p90RSK pathway and inhibits the proliferation of nasopharyngeal carcinoma cells in mouse tumor xenograft models
Source: Front Cell Infect Microbiol. 2023 Jan 4;12:1078504. doi: 10.3389/fcimb.2022.1078504 (PMC9850235; doi:10.3389/fcimb.2022.1078504)
Supplement: Supplementary file 1 [file DataSheet_1.docx]

**Supplementary materials**

**Journal:**

Frontiers in Cellular and Infection Microbiology

**Title:**

EBV LMP1-C terminal binding affibody molecule downregulates MEK/ERK/p90RSK pathway and inhibits the proliferation of nasopharyngeal carcinoma cells in mouse tumor xenograft models

**Authors**:

Yanru Guo *, Saidu Kamara*, Jing Zhang, He Wen, Maolin Zheng, Ying Liu, Luqi Zhou, Jun Chen, Shanli Zhu, Lifang Zhang

* These authors contributed equally to this work.

Institute of Molecular Virology and Immunology, Department of Microbiology and Immunology, School of Basic Medical Sciences, Wenzhou Medical University, Wenzhou 325035, Zhejiang, PR China.

Correspondence: Lifang Zhang ([wenzhouzlf@126.com](mailto:wenzhouzlf@126.com) ), +8613634286323

**Table S1. Primary antibodies used for Western blotting assay
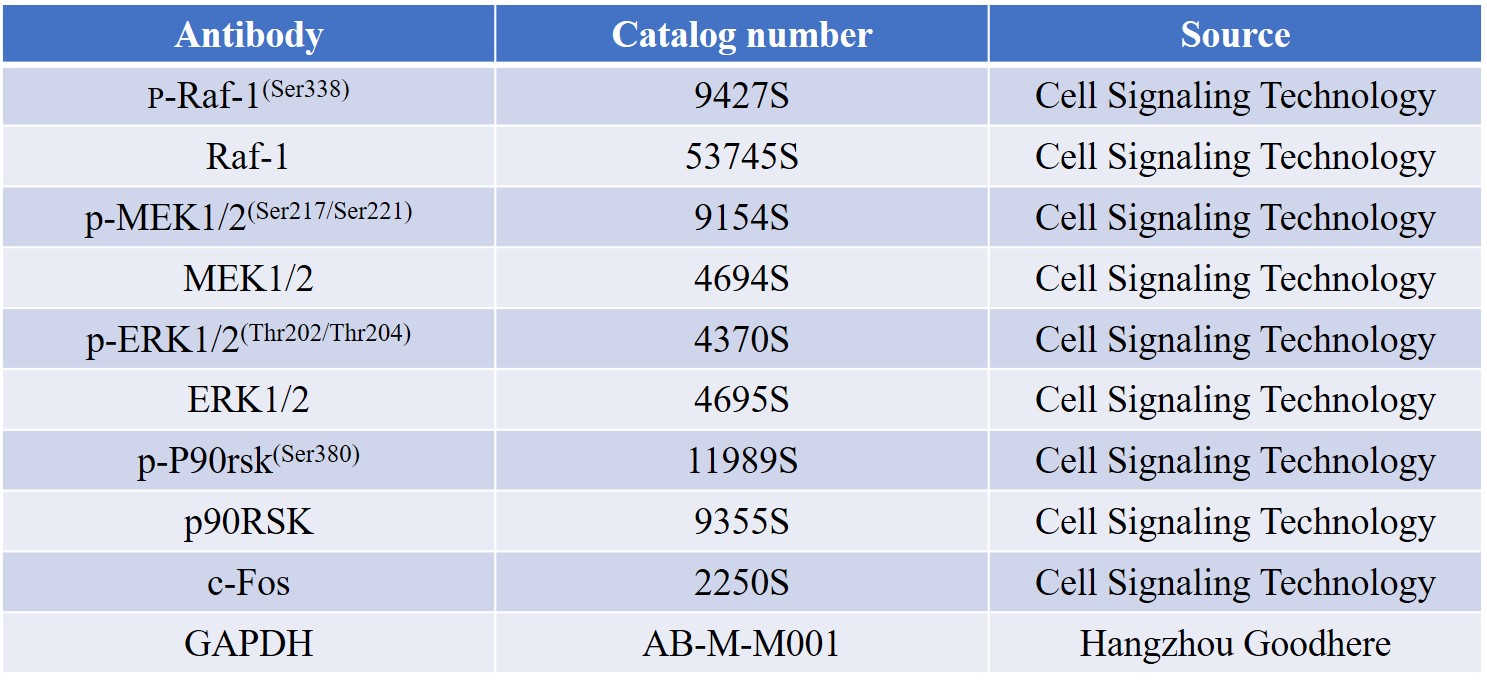
**

**Figure S1. (A)** C666-1 and **(B)** CNE-2Z cells were treated with different concentrations of Z_LMP1-C_ affibody for 72 h. IC50 values were calculated using graph pad prism.

**
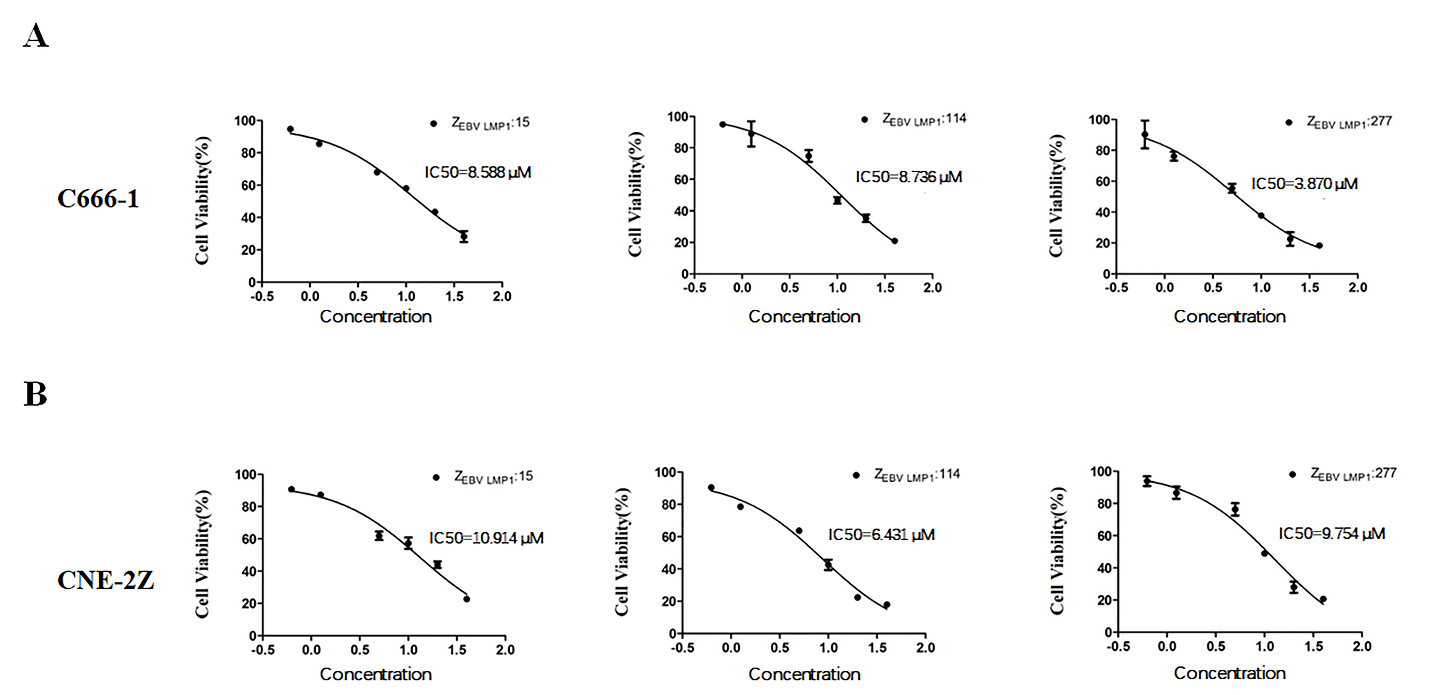
**

**Fig S2**. **Analysis of MEK/ERK/p90RSK pathway by Western blotting**. As shown in (**Fig. S2A**), Z_LMP1-C_277 treatment did not decrease phospho-Raf-1^(Ser338)^ in a concentration- and time-dependent manner in HNE-2 cell line. Western blotting showed that Z_LMP1-C_277 did not induced a reduction of phospho-MEK1/2 ^(Ser217/Ser221)^, phospho-ERK1/2^(Thr202/Thr204)^, phospho-p90RSK^(Ser380)^ and transcription factor c-Fos levels in HNE-2 cell line (**Fig. S2B**).

**
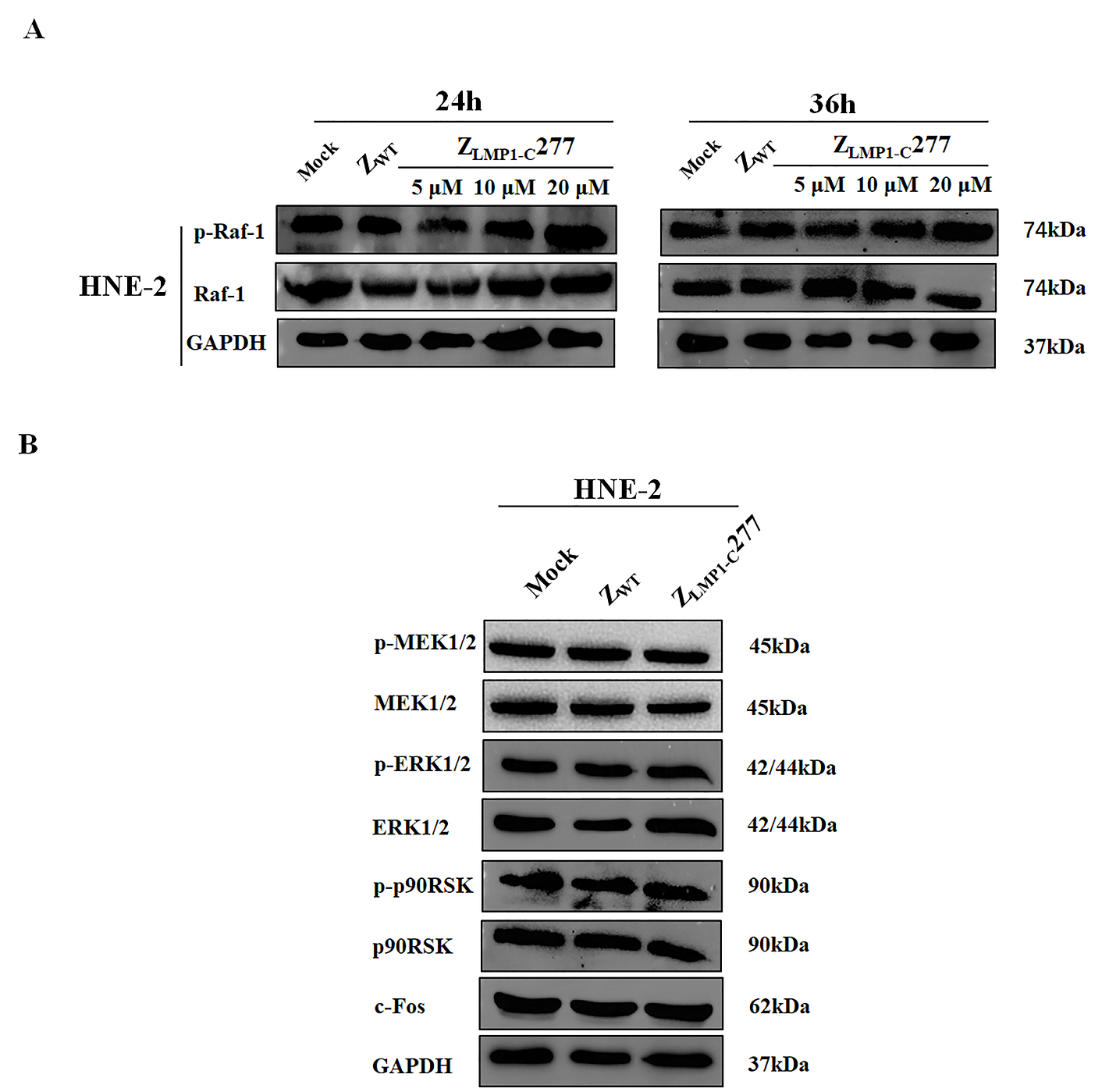
**
